# Looking down the road: development and distribution of a future syphilis vaccine

**DOI:** 10.1097/OLQ.0000000000002397

**Published:** 2026-08-21

**Authors:** Arlene C. Seña, Lorenzo Giacani, Caroline E. Cameron, Melissa J. Caimano, Michael Marks, Mathew A. Beale, Linda Grillova, Suzanne Day, Kimberly Workowski, Adele Schwartz-Benzaken, Kelly L. Hawley

**Affiliations:** 1 Department of Medicine, Division of Infectious Diseases, University of North Carolina, Chapel Hill, North Carolina, USA.; 2 Institute for Global Health and Infectious Diseases, University of North Carolina, Chapel Hill, North Carolina, USA.; 3 Department of Medicine, Division of Allergy and Infectious Diseases, University of Washington, Seattle, USA.; 4 Department of Global Health, University of Washington, Seattle, USA.; 5 Department of Biochemistry and Microbiology, University of Victoria, Victoria (BC) Canada.; 6 Departments of Pediatrics and, UConn Health, Farmington, Connecticut, USA.; 7 Department of Molecular Biology and Biophysics, UConn Health, Farmington, Connecticut, USA; 8 London School of Hygiene & Tropical Medicine, London, UK.; 9 Hospital for Tropical Diseases, University College London Hospital, London, UK; 10 Division of Infection and Immunity, University College London, London, UK; 11 Parasites and Microbes Programme, Wellcome Sanger Institute, Wellcome Genome Campus, Hinxton, UK.; 12 Department of Medicine, Emory University, Atlanta, Georgia, USA.; 13 AIDS Healthcare Foundation Brazil, Manaus, Brazil.; 14 Department of Immunology, UConn Health, Farmington, Connecticut, USA.; 15 Department of Research Connecticut Children’s Research Institute, Divisions of Research and Infectious Diseases, Connecticut Children’s, Hartford, Connecticut, USA.

**Keywords:** *Treponema pallidum*, syphilis, vaccine development

## Abstract

A symposium was held to explore critical topics in syphilis vaccine development, including identification of *Treponema pallidum* outer membrane proteins, genomic diversity, and considerations for future vaccine trials and implementation.

The World Health Organization (WHO) has identified vaccine development for syphilis as a sexually transmitted infection (STI) research priority. There were approximately 8 million new cases of syphilis among individuals aged 15 to 49 years old, and 700,000 cases of congenital syphilis reported in 2022.^1^ This alarming burden of disease underscores the need for a coordinated effort to accelerate effective syphilis vaccine development to help reverse rising infection rates.

In July 2025, an STI and HIV World Congress symposium was held in Montreal, Quebec, Canada, to explore critical areas in the development of a globally effective *Treponema pallidum* subspecies *pallidum* (*Tp*) vaccine. In addition to scientific presentations, an expert panel was convened to respond to key questions regarding syphilis vaccine trials and implementation. In this summary, we describe the following symposium topics: 1) *Tp* outer membrane proteins (OMPs) and development of a chimeric syphilis vaccine; 2) rational vaccinology and correlates of protection; 3) syphilis genomic diversity in Africa; 4) willingness to participate in a clinical vaccine trial; 5) potential challenges in clinical vaccine trials and implementation; 6) global considerations in vaccine implementation; and 7) bridging the academic-industry gap.

## *Tp* outer membrane proteins and development of a chimeric syphilis vaccine candidate

Prior rabbit experiments and clinical studies have supported the premise that prior *Tp* infection can confer partial (not sterilizing) immunity, and lead to attenuation of organism burden and clinical manifestations during subsequent infections.^2–3^ Following animal model experiments to understand the level of protection induced by experimental syphilis, the concept that protective immunity could be achieved by “artificial” *Tp* immunization surfaced in the late 1940s.^4^ Significant evidence has since accumulated that *Tp*-specific opsonic antibodies are the principal effectors of infection-induced protective immunity.^5^ This finding launched an ongoing quest to identify *Tp* surface-exposed OMPs, which comprise the most likely targets for opsonic antibodies and serve as optimal vaccine candidates.^5–7^ Freeze-fracture electron microscopy studies revealed a peculiar *Tp* outer membrane with a remarkable paucity of surface exposed proteins compared to conventional diderm bacteria.^8–9^ After syphilis research entered the genomics and proteomics era, the application of *in silico* prediction approaches to the *Tp* proteome led to the identification of proteins predicted to fold into β-barrels, the predominant hallmark of OMPs.^10–13^ Efforts to identify these rare putative OMPs have identified approximately 35 known or predicted OMPs in the *Tp* proteome.^11^

As the number of putative *Tp* OMPs increases, so has the inventory of predicted surface-exposed extracellular loops (ECLs) that these proteins harbor. Thus, investigators are now facing the challenge of identifying which ECLs are the most relevant immunogens to induce the functional immunity necessary for bacterial clearance. Despite the limited number of OMPs discovered to date, some putative OMPs contain a relatively large number of predicted ECLs. For example, TP0326 (BamA) and TP0151 (LptD), are predicted to have eight and thirteen ECLs, respectively. Even smaller proteins, like TP0126 or TP0733, or TP0292 (one of the *TPA* OmpW homologs; Giacani *et al*., unpublished) contribute to the total *Tp* ECL repertoire with four to six ECLs, respectively.^13–14^ Therefore, understanding the antigenic properties of individual ECLs during natural and experimental infection, or following immunization, has become a pivotal effort in vaccine development.

In parallel with this research focus, the complementary protein engineering approach of chimeric scaffold development has arisen, where chimeric antigens are built using scaffold platforms that can accommodate multiple epitopes from ECLs known or predicted to be associated with protective immunity. This strategy compensates for the low density of protein targets on the *Tp* host-facing surface, as well as our minimal knowledge of regulation of OMP expression during infection, which currently is limited to a few OMPs^15–16^ This approach also allows for multiple *Tp* proteins to be included in a vaccine to achieve protective efficacy.

Drs. Giacani and Cameron discussed ongoing work that uses a two-pronged approach to vaccine design targeting the localized and disseminated stages of *Tp* infection.^17–19^ Dr. Cameron and her team developed the concept of using the stable, soluble, and protein-engineering malleable TP0751 protein as a scaffold and replaced multiple loops of this known host-targeting adhesin^10,20^ with ECLs from several *Tp* OMPs (Figure 1). This included epitopes from the TprC (TP0117) protein, partially shared with TprD/D2 (TP0131), as well as TprK (TP0897) epitopes that were identified by Dr. Giacani’s group, and shown to significantly attenuate chancre formation in the intradermal challenge rabbit model.^19^ In its current iteration, the TP0751-based chimeric construct comprises nine ECLs from seven *Tp* OMPs. Additionally, because TP0751 is a vascular adhesin that contributes to endothelial cell attachment and *Tp* dissemination, the immunity generated against the TP0751 “functional scaffolding” also contributes to the vaccine’s protective ability.^20–21^ Drs. Cameron and Giacani discussed pre-clinical experiments to assess the efficacy of the latest chimera design. They also discussed the importance of sourcing effective commercially available or soon-to-be-available adjuvants to induce a strong T_H_1 response, since opsonic antibodies and the release of interferon-γ both function to stimulate macrophages to phagocytose and clear treponemes through opsonophagocytosis.^22–23^

## Rational vaccinology and correlates of protection

Based on the hypothesis that antibodies to *Tp*’s rare OMPs are the principal effectors of infection-induced immunity, Dr. Caimano presented her group’s multidisciplinary “learning from nature” approach to syphilis vaccine development. Using *Pyrococcus furiosus* thioredoxin (*Pf*Trx) scaffolds displaying *Tp* ECLs (Figure 2A) to screen sera from immune rabbits and humans with primary and secondary syphilis, they identified multiple ECLs that are strongly immunogenic during natural infection.^24–25^ ECLs were selected for investigation based on sequence conservation across *Tp* strains circulating in geographically distinct sites.^26–29^ Highly promising targets included ECL4 from BamA (TP0326) and multiple ECLs from the FadL-like orthologs TP0856, TP0858 and TP0865.^25,30–31^ Immunization of rabbits with individual *Pf*Trx scaffolded ECLs elicited high titers of loop-specific IgG, confirming that these regions retain immunogenicity.^29–30^ Importantly, anti-ECL antibodies generated using *Pf*Trx-ECL scaffolds promoted phagocytic uptake and spirochete killing at levels comparable to those observed with immune rabbit serum (IRS).

Building on recent advances in *Tp in vitro* cultivation,^31^ Dr. Caimano reported on the development of *in vitro* assays to systemically compare the functionality of ECL-specific antisera with that of IRS. Using a *Tp* strain expressing green fluorescent protein,^32^ Dr. Caimano, Dr. Hawley and colleagues demonstrated that ECL-specific antibodies against BamA ECL4, TP0858 ECL2 and ECL4, and TP0865 ECL3 exert their bactericidal effect, in part, by disrupting *Tp*’s fragile outer membrane.^30,32^ Intradermal challenge of rabbits using treponemes pre-incubated with ECL-specific antibodies resulted in absent or transient lesions and substantially lower bacterial burdens.^30^ Although a formal correlate of protection for syphilis has yet to be established, the combined readouts of these assays provide quantitative functional surrogates lacking in the field, enabling a rational evaluation of vaccine candidates to be prioritized. Despite the promise of single-ECL approaches, the low density of *Tp* OMPs and potential differences in OMP expression during infection and inter-individual variability in immune responses suggest that an effective vaccine likely requires a multivalent strategy. Scaffolds capable of displaying multiple ECLs from the same, or different, *Tp* OMPs represent a logical next step, enabling coordinated presentation of multiple protective epitopes within a single scaffold. Towards this end, Dr. Caimano reported on their development of multivalent scaffolds based on the loopless C-lobe of *Neisseria meningitidis* transferrin-binding protein B (LCL-TbpB)^33–34^ and a truncated version of *Escherichia coli* OmpA that lacks the periplasmic protein domain,^35^ each capable of displaying up to four ECLs (Figure 2B). Both chimeric scaffolds elicited robust antibody responses against multiple ECLs without compromising reactivity to individual components, achieving ECL-specific titers comparable to or greater than those observed with the corresponding *Pf*Trx-ECL scaffolds (unpublished data). Comparisons of antibody titers and functionality of sera from rabbits immunized with single- versus multi-ECL scaffolds are underway.

## Syphilis genomic diversity in Africa

Understanding the genomic diversity of *Tp* strains is important to syphilis vaccine development. Whole genome sequencing by means of hybrid capture enriched metagenomics using RNA/DNA probes has emerged as the dominant method for assessing the genomic diversity of *Tp* strains circulating worldwide.^36^ These approaches provide a step change in resolution compared to traditional multi-locus-sequence-typing approaches. Current approaches have predominantly utilized residual DNA from specimens collected from individuals who present for care. This reliance on residual clinical samples results in biases towards populations that undergo regular testing (*e.g*., gay, bisexual and other men who have sex with men [GBMSM]) and to regions where testing is available (*e.g*., high income countries [HICs]). The overwhelming majority of *Tp* sequence data comes from GBMSM patients from the Global North. Consequently, the regions with the highest burden of disease (*e.g*., Africa) and the populations most at risk (*e.g*., women of childbearing age at risk of congenital syphilis) remain poorly sampled, representing less than 15% of available *Tp* genomes.^37^

Dr. Marks and his team discussed how *Tp* sequence data is now considered critical to understand global antigenic diversity within candidate vaccine targets,^27^ and ensure that vaccines will be deployable globally. Due to the limitations of sampling from care-seeking individuals, improving representation among genomic datasets requires prospective studies. Despite the fact that the highest burden of syphilis morbidity and mortality are in African regions,^38^ they have to date been underrepresented in global sequencing efforts, with the majority of published genomic data restricted to historic samples from Madagascar^39^ and a prospective study in Malawi.^27^

Epidemiological analysis of genomic data from HICs demonstrates the GBMSM syphilis pandemic is dominated by a small number of globally circulating lineages, consistent with inter-connected sexual networks.^40^ To address the knowledge gap from low- and middle-income countries (LMICs), Dr. Marks’ group established collaborations with multiple countries across East and Southern Africa, facilitating prospective patient recruitment and sample collection. This has resulted in generation of the first genomes from Botswana, Uganda and a significant expansion in the number of genomes available from Zimbabwe and South Africa. Contextual analysis of these and other genomes obtained from Africa^37^ revealed that in contrast to the global picture of a small number of shared sublineages, most syphilis circulating in Africa is caused by local lineages that have not been identified elsewhere. Notably, in South Africa, there has been an increasing proportion of global lineages relative to local lineages in recent years, suggesting changes in the structure and international connections of the sexual network. This work emphasizes the need for further surveillance in LMICs, which may have substantially different genomic population structures and novel diversity. Moreover, the finding that population structure can change over time has implications for the long-term utility of vaccines developed against a subset of global diversity.

To complement core-genome analyses for assessing transmission and evolution, investigators have focused on the analysis of *Tp* OMPs as primary vaccine candidates.^25–27, 41^ This requires different methodological approaches, combining wet and dry lab techniques. Some *Tp* OMPs are variable or hypervariable and contain complex recombination and repetitive sequences that make them difficult to sequence and analyze using standard methods. The researchers at the Wellcome Sanger Institute described their analyses of 38 membrane-associated proteins, including six newly proposed OMPs, using a global dataset of 1,370 genomes collected between 2000 and 2023. Amino acid replacements were assessed only in residues predicted to be exposed on the protein surface, while substitutions in membrane-buried residues were excluded from the analysis. This approach led to the identification of 15 proteins with highly conserved surface residues (unpublished data). The strong conservation of these *Tp* proteins across six continents, including six African countries, makes them promising targets for a universal vaccine. While conservation suggests evolutionary stability and broad coverage across strains, it may also reflect limited host-pathogen interaction. Therefore, integrating these findings with immunological data is essential to identify targets that elicit a protective immune response.

## Willingness to participate in a clinical vaccine trial

In anticipation of future syphilis vaccine trials, there is a need to understand factors that may influence trial participation decision-making. Dr. Day discussed an exploratory qualitative study conducted in Malawi to identify barriers and facilitators of syphilis vaccine trial participation, with the goal of surfacing priorities for community engagement in preparation for trials. The study used a semi-structured interview guide and visual aids to explore the views of Malawians with recent STI diagnosis regarding syphilis vaccine trial participation. Dr. Day discussed findings which echoed her prior U.S. study among people with recent STI diagnosis, men who have sex with men (MSM), and people living with HIV (PLWH).^42^ For example, altruistic desires to help fellow Malawians through the process of scientific discovery was a notable motivator of willingness to participate in vaccine trials. However, barriers to participation surfaced that were unique to the Malawian context, indicating that preparation for vaccine studies must include specific considerations that may hinder trial participation. Previous research has shown that failure to consider local concerns can lead to mistrust of clinical research,^43^ and can even result in vaccine trial shut-down.^44–46^ Additional investigations are thus warranted to identify the concerns, needs, and priorities of potential participants and communities prior to trial implementation in order to minimize social and ethical impediments to syphilis vaccine research and vaccine acceptability.

## Potential challenges in clinical vaccine trials and implementation

The vaccine type, efficacy, duration of protection, selection of key populations, correlates of protection, and impacts on clinical and public health practice are important factors to consider in future syphilis vaccine trials. Dr. Workowski discussed these factors and other considerations (Table 1), including the potential effects of vaccination on syphilis clinical presentation and serologic diagnosis (among persons with vaccine breakthrough infections). Prior vaccination and partial protection could potentially alter the manifestations of acquired syphilis. Syphilis diagnosis would thus be challenging in a vaccine trial since currently available nontreponemal (lipoidal antigen) and treponemal antibody tests would not be able to differentiate between new infections, prior infections or vaccinated status. This underscores the importance of new syphilis diagnostics that are both highly sensitive and specific for the detection of active infection with *Tp* (rather than the immune response), in order to guide clinical management. In addition to the individual-level effects of syphilis vaccination, public health issues need to be considered including how to address the stigma and distrust surrounding syphilis as a result of the egregious Tuskegee trials, and vaccine hesitancy across different populations. Lastly, current challenges in the state of STI research funding in the U.S. have broad ramifications globally, as investigators strive to advance syphilis research from translational science to future clinical trials.

## Global considerations in vaccine implementation

There are additional challenges to identifying and prioritizing key populations for syphilis vaccine implementation worldwide. Dr. Benzaken emphasized that a syphilis vaccine should be suitable for use in pregnant women, their sexual partners, and as a secondary prevention measure for reinfections. The vaccine should be available to PLWH, and those at risk for other STIs including MSM, sex workers, and people who inject drugs, who often experience barriers to vaccination due to stigma or discrimination. Vaccine allocation must prioritize high-burden countries, including nations in sub-Saharan Africa and Latin America where congenital syphilis rates are high. Integration with existing health programs (*e.g*. antenatal care, HIV and PrEP clinics, STI clinics) will be critical to maximize reach and efficiency.

When considering the introduction of a syphilis vaccine in LMICs, Dr. Benzaken discussed critical characteristics that must be addressed to ensure maximum accessibility and impact. Ideally, the vaccine should require single-dose administration, which is important for populations that are difficult to access. The vaccine should demonstrate thermostability, with a preference for room temperature or 2–8°C storage, making it compatible with existing cold chain systems. Additionally, a long shelf life (preferably more than two years) will support efficient distribution and reduce waste. Robust scientific evidence will be necessary to facilitate WHO prequalification and support national regulatory approvals. Cost is another crucial factor; the vaccine must be affordable, with a price point comparable to other vaccines, such as the human papilloma virus vaccine. Dr. Benzaken stated that procurement should be facilitated by mechanisms like the Pan American Health Organization or the African Centers for Disease Control’s pooled procurement systems, which help improve vaccine accessibility through centralized purchasing. Planning for syphilis vaccine deployment should focus on decentralized and scaled production, with regional manufacturing capacity established and expanded in advance.

## Bridging the academic-industry gap

Lastly, Dr. Carolyn Deal from the National Institutes of Health discussed the importance of bridging the *Tp* translational vaccine research to potential industry partners early in the developmental process. She emphasized that analyses of the economic impact of syphilis and congenital syphilis on public health should be performed to justify the need for vaccine development. The WHO’s roadmap for STI vaccine development has included how to encourage investment and make a business case for vaccine manufacturers by outlining disease burden epidemiology and modeling vaccine impact to facilitate defining preferred product characteristics.^47^ Preferred product characteristics would be necessary to reflect WHO guidance on the desired vaccine parameters that would meet priority public health goals, especially for LMICs. Modeling studies would be informative to evaluate different vaccination strategies in both HICs and LMICs, depending on key populations for vaccination, vaccine efficacy and duration of protection. For example, an integrated transmission-dynamic, health-economic modeling analysis for the *Neisseria meningitidis* (meningococcus) serogroup B (4CMenB) vaccine demonstrated that vaccination of individuals according to self-reported behavior in England would achieve the fastest and largest impact, but at high cost relative to the gonorrhea cases to be averted.^48^

## Conclusions

The past decade has witnessed significant advances in translational research involving potential *Tp* vaccine candidates and vaccine approaches, accompanied by an increasing number of studies describing *Tp*’s genomic diversity and conserved proteins across strains. Studies involving syphilis vaccine acceptability are underway, and experts are beginning to consider key populations, vaccine trial design and vaccination strategies. A syphilis meeting of scientists, clinicians and public health leaders worldwide held in Shanghai, China in October 2025 involved additional presentations on *Tp* vaccine development,^49–51^ highlighting the ongoing collaborations to address the global syphilis epidemic. Although many challenges remain and vaccine implementation is many years from fruition, there is a promising path ahead. Robust multi-disciplinary research, funding mechanisms, and strategic planning must continue in order to achieve a better future in which syphilis vaccine development and implementation can be realized.

## Figures and Tables

**Figure 1:**
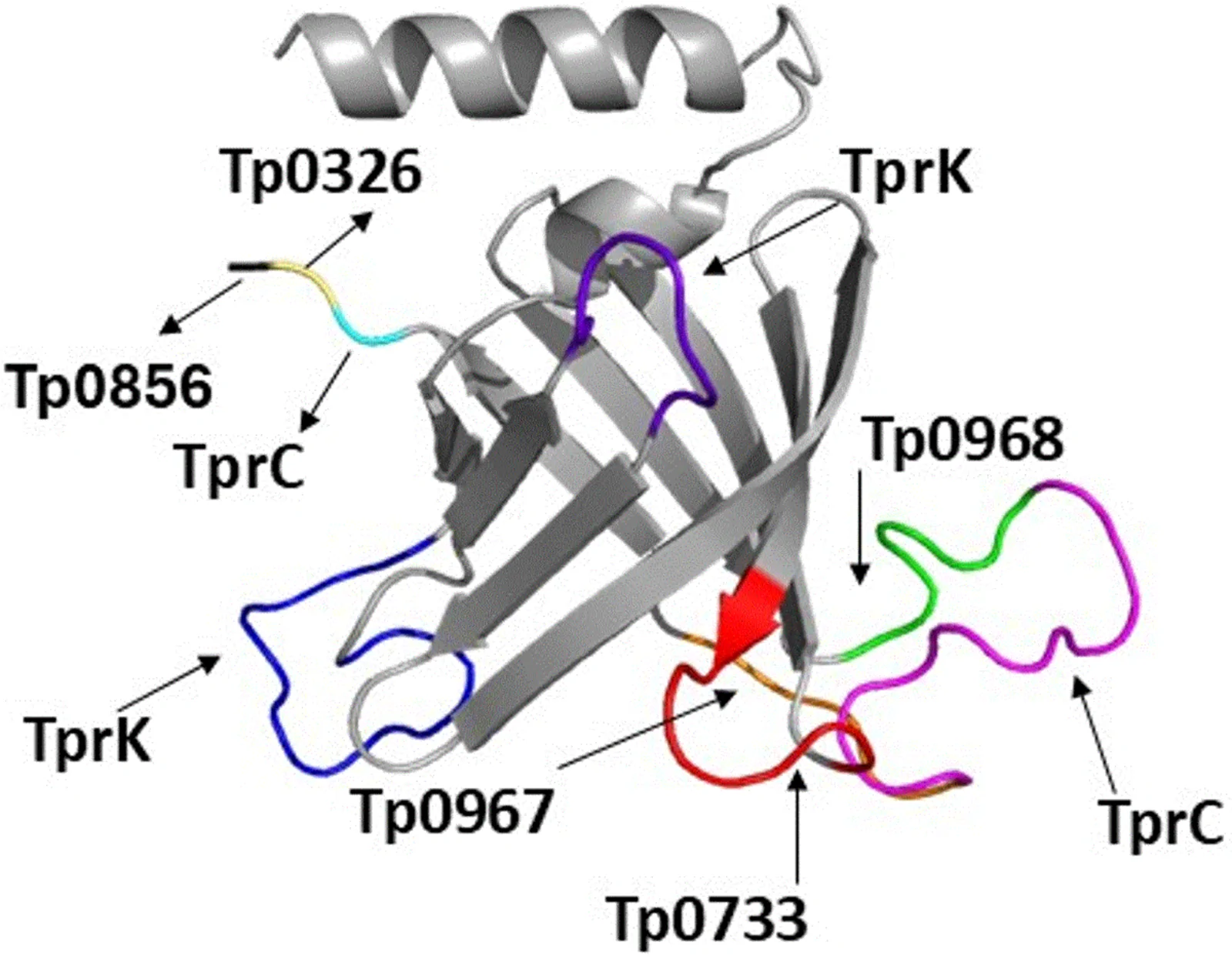
One of the TP0751-based chimeric vaccine constructs being pursued as a syphilis subunit vaccine candidate. The TP0751-based scaffold platform is amenable to protein engineering with replacement of the TP0751 flexible loop regions with epitopes from other *Tp* OMPs. The platform is compatible with manufacturing requirements; the chimera vaccine construct is soluble, stable, and can be expressed at high yield with endotoxin levels below the FDA threshold of 5 Endotoxin Units (EU)/kg of body weight.48 The chimera version shown includes nine independent epitopes from seven different *Tp* proteins inserted into the TP0751-based scaffold.

**Figure 2.**
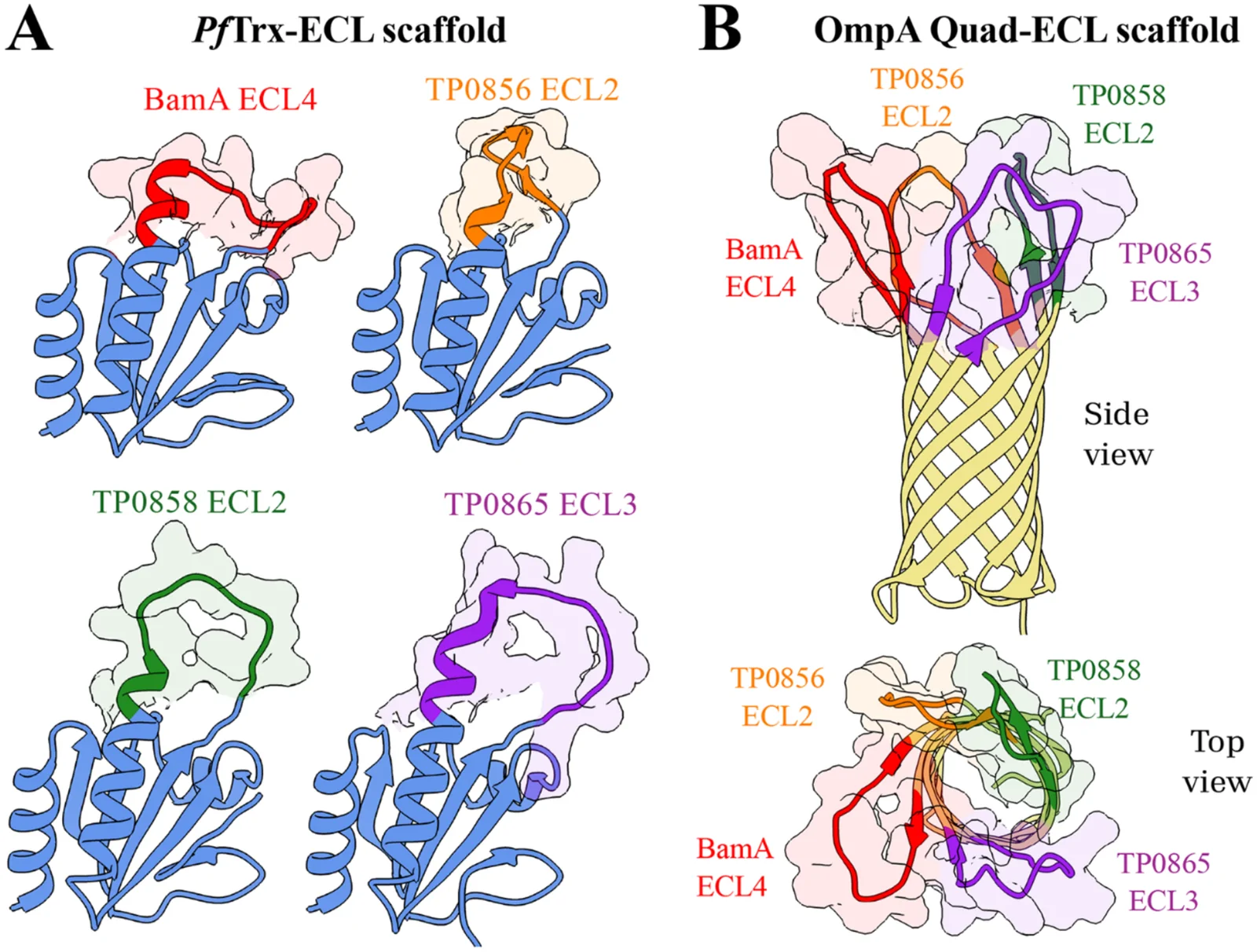
Recombinant protein scaffolds displaying *Treponema pallidum* OMP extracellular loops. **A**. *Pyrococcus furiosus* thioredoxin (*Pf*Trx; blue) scaffolds displaying individual ECLs from BamA (red) and the FadL-like orthologs TP0856 (orange), TP0858 (green) and TP0865 (purple). **B**. *Escherichia coli* OmpA β-barrel scaffold displaying the four OMP-ECLs shown in A.

**Table 1:** Considerations for future syphilis vaccine trials and implementation

Parameter	Key questions
Vaccine type	Can the type of vaccine (*e.g*. subunit, multivalent, mRNA, outer membrane vesicles) demonstrate effectiveness given the global diversity of strains, based on *T. pallidum* proteins and extracellular loops?
Vaccine indication	Will the vaccine be able to prevent or attenuate *T. pallidum* infection, or provide partial or sterilizing immunity?
	Will there be a role for the vaccine as post exposure prophylaxis?
	Would marketing considerations focus on adult syphilis or congenital syphilis (protection of key populations versus infants)?
Target populations	Who should be the initial target populations for rollout of vaccine?
	How would we ensure diversity and access to vaccinations?
	What type of community engagement is necessary for the target population?
Vaccine efficacy	What are the correlates of a protective immune response?
	Would there be differences in immune response among persons who are immunocompromised (*e.g*. persons with HIV infection with or without antiretroviral therapy, defects in T or B cells, pregnancy)?
	Would the immune response to vaccination be similar in persons with no prior history of syphilis versus those with previous infection?
Duration of protection	Will a single dose vaccination be required or multiple dose vaccine schedule? Will the vaccine schedule differ in those with previous syphilis infection?
Vaccine delivery strategy	What would be the route of vaccine administration (*e.g*. intramuscular, subcutaneous, intradermal)? Does the route of administration effect vaccine efficacy?
	What would be the cold chain requirements for vaccine storage?
	Would there be insurance coverage, or lowered cost of vaccines in resource-poor countries?
Clinical considerations	If the vaccine provides only partial protection, will the clinical presentation of syphilis be attenuated?
	Will vaccine breakthrough infections be more challenging to identify clinically, given the complexity of syphilis?
	What are the clinical implications of vaccines on transmission risk and management decisions?
	Will vaccination affect the consequences of severe infection (*e.g*. central nervous system infection, cardiovascular syphilis)?
	Would syphilis serologies (nontreponemal and treponemal antibodies) be able to distinguish between natural infection, prior infection vs vaccine breakthrough infections?
	What would be the effect of vaccination on serologic response to subsequent syphilis infection? Does this have implications for clinical management?
Public health considerations	How should we address the stigma surrounding syphilis (from healthcare providers, patients, the public) as a result of the Tuskegee trials?
	How should we use previous STI vaccine messaging and delivery (*e.g*. human papilloma virus, Mpox) to minimize vaccine hesitancy and address geographic or population differences in vaccine acceptance?
	What would be the effects of syphilis vaccination on the public health work force (*e.g*. disease intervention specialists conducting partner notification)?
	Would the vaccine have effects on individual sexual behaviors?
	If adequate vaccination rates are achieved, will this have an effect on use of doxycycline postexposure prophylaxis or benzathine penicillin production?
Vaccine research	Can syphilis vaccination decrease syphilis rates over time? (modeling)
	What is the cost-effectiveness of future vaccination strategies? (modeling)
